# Decreased small-world functional network connectivity and clustering across resting state networks in schizophrenia: an fMRI classification tutorial

**DOI:** 10.3389/fnhum.2013.00520

**Published:** 2013-09-02

**Authors:** Ariana Anderson, Mark S. Cohen

**Affiliations:** Department of Psychiatry and Biobehavioral Sciences, Center for Cognitive Neuroscience, University of California Los AngelesLos Angeles, CA, USA

**Keywords:** fMRI, classification, functional network connectivity, SVM, independent component analysis, *R*, Schizophrenia, small-world

## Abstract

Functional network connectivity (FNC) is a method of analyzing the temporal relationship of anatomical brain components, comparing the synchronicity between patient groups or conditions. We use functional-connectivity measures between independent components to classify between Schizophrenia patients and healthy controls during resting-state. Connectivity is measured using a variety of graph-theoretic connectivity measures such as graph density, average path length, and small-worldness. The Schizophrenia patients showed significantly less clustering (transitivity) among components than healthy controls (*p* < 0.05, corrected) with networks less likely to be connected, and also showed lower small-world connectivity than healthy controls. Using only these connectivity measures, an SVM classifier (without parameter tuning) could discriminate between Schizophrenia patients and healthy controls with 65% accuracy, compared to 51% chance. This implies that the global functional connectivity between resting-state networks is altered in Schizophrenia, with networks more likely to be disconnected and behave dissimilarly for diseased patients. We present this research finding as a tutorial using the publicly available COBRE dataset of 146 Schizophrenia patients and healthy controls, provided as part of the 1000 Functional Connectomes Project. We demonstrate preprocessing, using independent component analysis (ICA) to nominate networks, computing graph-theoretic connectivity measures, and finally using these connectivity measures to either classify between patient groups or assess between-group differences using formal hypothesis testing. All necessary code is provided for both running command-line FSL preprocessing, and for computing all statistical measures and SVM classification within R. Collectively, this work presents not just findings of diminished FNC among resting-state networks in Schizophrenia, but also a practical connectivity tutorial.

## 1. Introduction

Functional Magnetic Resonance Imaging (fMRI) is a four-dimensional medical imaging modality that captures changes in blood oxygenation over time, an indirect measure of neuronal activation. Because fMRI scans are large, they are stored in specialized formats that make their direct access and manipulation difficult. Statistical analyses are therefore limited to the software the neuroscientist is able to use; pre-made routines are available to perform general analyses such as linear models, but the techniques and consequently the hypotheses that can be evaluated by them are limited and inflexible. Analyses are dependent upon the ability to create programs that not only can access directly subsets of the data, but also can be tailored to unique statistical analysis based on *a priori* hypotheses of the underlying neurological disorders.

An increasing focus is the classification of either mental disorders or states based on the fMRI signal variations within and among brain networks. One method of accomplishing this is through measurements of functional network connectivity (FNC), which infers differences in temporal brain connectivity that may depend on a disease or mental state (Biswal et al., 1995; van de Ven et al., 2004). FNC investigates temporal connectivity differences among either anatomical brain regions or functionally defined networks. Herein, we present a tutorial to perform FNC in *R* which can be altered easily for a unique hypothesis or dataset (Tabelow et al., 2011; R Development Core Team, 2012).

The methods we discuss here closely follows those presented in Anderson et al. (2010), which describes in full the motivation for, and findings of, using brain connectivity measures to classify between Schizophrenia patients and normal controls during rest. We demonstrate this procedure on a recently released dataset, publicly available for download at http://fcon_1000.projects.nitrc.org/indi/retro/cobre.html and studied previously in Calhoun et al. (2011), Hanlon et al. (2011), Mayer et al. (2012). This dataset, which we will refer to as the COBRE data, consists of 72 patients with Schizophrenia and 74 healthy controls, ranging in age from 18 to 65 years old. A full demographic table is provided in Table 1.

**Table 1:** **COBRE FCON-1000 data demographics**.

	**N**	**Age (*SD*)**	**% Female**	**% Right-Handed**
Schizophrenia	72	38.16 (13.89)	0.19	0.83
Patients	74	35.82 (11.58)	0.31	0.96

The code contained in this article is available through the Neuroimaging Informatics Tools and Resources Clearinghouse (NITRC) at http://www.nitrc.org/projects/fmriclassify/. NITRC is an NIH-sponsored project to categorize, compare, rate and distribute software and data, created by and for neuroimaging researchers. It contains both stand-alone programs and code snippets such as this project. Its usefulness is quite evident given the redundancies in research, where many labs develop independently routines to perform similar analysis techniques such as functional connectivity analysis. It is also useful for determining reproducibility, as users can test another's analysis on their own data to see if similar results are reached. This is particularly appropriate in fMRI analysis, where conclusions are often reached on quite small sample sizes since data are costly and difficult to obtain. The reader is encouraged to download and modify this code snippet from the NITRC website.

We demonstrate this analysis using preprocessing in FSL, which performs brain extraction [bet (Smith, 2002)] to remove non-brain tissue, motion-correction mcflirt (Jenkinson et al., 2002) to correct for subject movement within the scan, and ICA using melodic (Smith et al., 2004) with automatic component estimation. A full FSL tutorial is available at http://fsl.fmrib.ox.ac.uk/fslcourse/. We use independent component analysis (ICA) to identify networks within each patient and calculate properties of their temporal-connectivity, demonstrating this within FSL, implemented as “MELODIC”, and within *R*. Using packages **vegan** (Oksanen et al., 2011) and **AnalyzeFMRI** (Bordier et al., 2009), we extract possible neurological networks and define distances among them as functional connectivity measures. This distance matrix is then converted into a graph structure, and properties of these connectivity graphs are computed using **igraph** (Csardi and Nepusz, 2006). We use this connectivity for classification with the Support Vector Machines (SVM) algorithm in the package **e1071** (Dimitriadou et al., 2010).

Because this analysis is heavily computational, we also demonstrate how to perform this same process in parallel using the package **parallel** (R Development Core Team, 2012). The ability to code this in *R* with minimal function calls, or changing of the original code, allows users to implement and test computationally intensive analyses efficiently and simply. Parallel computing is a specialized topic, and many researchers are uninterested in learning methods such as MPI to implement their analyses, as troubleshooting can often take as long as the time saved by running in parallel. Because of this, we demonstrate calling fork clusters within *R* to perform parallel analysis, without making major revisions to the code already created to run in serial. This supplementary section is listed in the *Appendix*. We additionally demonstrate in the *Appendix* using R to access fMRI data, including how to perform ICA using the package **AnalyzefMRI** (Bordier et al., 2009).

We begin with a description of our approach, and follow with an applications section where we provide and discuss the code necessary to accomplish these methods. In this tutorial we assume the reader has no specific knowledge of *R*, but does have general knowledge of basic programming techniques. An *R* tutorial is available at http://cran.r-project.org/doc/manuals/R-intro.html. We hard-code as little as possible to ensure minimal changes for a new users' analysis. As this analysis focuses on connectivity within subjects, spatial alignment across subjects is not necessary, although procedures such as motion correction and temporal filtering may be performed beforehand if desired. The **AnalyzeFMRI**, **vegan**, **igraph**, and **e1071** packages are used along with their dependencies, and must be pre-installed. These packages are available at http://cran.r-project.org/web/packages. The package **parallel** is a base package installed already within the latest *R* release. As the bulk of this code is constructed to classify between distance matrices, these routines can be adapted easily for a region of interest (ROI) analysis where distances are sought not between independent components, but instead between ROIs. More generally, these methods are applicable to longitudinal data analysis where the temporal correlations among units are indicative of a state or condition. Collectively, this article demonstrates code that can be adapted easily to new data for determining if functional connectivity differences exist between groups of fMRI scans, and is meant to serve as a bridge between neuroscientists interested in performing their own connectivity/classification analysis, and statisticians interested in seeing these methods applied to real-world data.

## 2. Background

### 2.1. Overview of fMRI

Function magnetic resonance imaging is a modality that measures brain activity over time. The fMRI Blood Oxygen Level Dependent (BOLD) signal is an indirect reflection of neuronal activity captured during an fMRI scan, and analysis is performed under the assumption that neuronal activity coincides with increased blood flow. The blood flow increase in response to neuronal activity is known as the hemodynamic response (Kim et al., 1999). When activation occurs within a region, oxygenated hemoglobin flows to that area to increase the local oxygen concentration. Deoxyhemoglobin has a faster MR signal decay rate (T2^*^) than oxyhemoglobin (Cohen and Bookheimer, 1994), so the signal from well-oxygenated regions results in a stronger MR signal intensity than areas lacking the increased blood flow. Areas with increased neuronal activity therefore give off a greater MRI signal, which indicates potential neural activity.

The four-dimensional fMRI picture can be used to discover anatomical regions specific to certain tasks such as language processing (Bookheimer, 2002), face recognition (Gauthier et al., 1999), or even to diagnose regional impairment specific to cognitive disorders such as Alzheimers disease, traumatic brain injury (TBI), or schizophrenia (Ford et al., 2003; Anderson et al., 2010). Such studies typically analyze regional blood flow to establish areas active during a task, or to compare regional blood oxygenation levels between two groups, such as Alzheimer's patients and normal controls, to find localized variation that could be the cause of cognitive impairment.

FNC is used to test the hypotheses that synchronicity across anatomically-defined brain regions or functionally-hypothesized networks are different, because of age, disease, or the task being performed. Connectivity differences are thought to underly many disorders such as autism (Koshino et al., 2005) and schizophrenia (Liang et al., 2006). More generally, temporal connectivity has been used to explore directed influences between neuronal populations in fMRI data (Roebroeck et al., 2005) using Granger causality, and to examine differences between schizophrenia patients compared to normal controls (Garrity et al., 2007; Jafri et al., 2008; Anderson et al., 2010; Yu et al., 2011) using cross-correlation measures. Within Schizophrenia, disrupted small-world properties were found compared to healthy controls among 90 cortical and subcortical regions (Liu et al., 2008). Increased regional functional connectivity in the 0.06–0.125 Hz interval were found in Schizophrenia, along with decreased strength by Bassett et al. (2008). Within the default mode network, abnormally high functional connectivity and altered temporal frequency have been found (Garrity et al., 2007; Whitfield-Gabrieli et al., 2009). Schizophrenia patients had higher correlations among seven selected resting-state networks than healthy controls (Jafri et al., 2008), and different topological measures were found between resting-state networks identified by group-ICA (Yu et al., 2011). Collectively, these works and others propose that within Schizophrenia, functional connectivity measures can be used to identify traits that are characteristic of the disease itself.

Because connectivity depends on how networks or regions are defined, and how the graphical properties of regions may be measured (Toppi et al., 2012; Zalesky et al., 2012), it is vital for researchers to be able to tailor connectivity analysis to their own data, to allow pre-existing knowledge or certain hypothesis to be tested. For example, Sato et al. (2010), implemented functional connectivity analysis among regions of interest, while Chu et al. (2011), analyzed connectivity among individual voxels. Similarly, Yu et al. (2011) analyzed FNC among group-ICA components, while we analyzed FNC among within-subject ICA components (Anderson et al., 2010).

### 2.2. fMRI classification

The primary challenge of fMRI classification is the abundance of observations within a single scan, many of which are correlated strongly both in space and time. Although many of these voxels will be empty, they are not systematically empty across subjects as a result of differences in brain size and shape. Because many of these datapoints are redundant, dimension reduction techniques are used by creating statistical summaries of individual voxels (*t-tests*, correlation tests), isolating “regions of interests” (ROI) or neural hotspots on which discrimination could be performed, or implementing classical dimension reduction methods such as principal components analysis (PCA) to decompose the entire scan into orthogonal signal sources over time. Newer methods such as ICA (Hyvärinen and Oja, 2000) and Sparse Component Analysis (Georgiev et al., 2005) mimic the approach of PCA into decomposing the scan into a limited number of spatial networks operating over time, but alternatively impose assumptions such as statistical independence or sparsity to estimate the underlying signal sources.

Once the dimension reduction and feature extraction steps are complete, the reduced data are fed into classifiers such as SVM, random forests, and boosting algorithms. These classification techniques have been used previously to discriminate between Positron Emission Tomography (PET) scans of HIV positive and healthy individuals (Liow et al., 2000), to detect deceptive individuals within a group using fMRI (Lee et al., 2002; Fan et al., 2006), to separate drug-addicted patients from healthy controls using fMRI scans (Zhang and Samaras, 2005), and to discriminate between patients with Alzheimer's, schizophrenia, and TBI and healthy controls using fMRI scans (Ford et al., 2003).

In “leave one out” cross-validation, these classifiers often achieve around 90% accuracy, but because methods are constructed uniquely for each dataset they are difficult to validate across different patient groups, or even within the same patient group but with a new population. These studies are often performed on excessively small samples (*n* ≈ 20). The reproducibility of such findings are often unverified, leaving open the criticism that superior classification accuracy is due to mere chance or model-mining, instead of underlying functional or anatomical differences between patient groups. It can be difficult to pool data taken across laboratories, because the scan parameters, resolution, and imaging sequences would have to be nearly equivalent. Because of this, the ability to evaluate models on different datasets would increase confidence in results, since the models acting on the same patient group should produce identical results, holding the scan environment constant.

### 2.3. R for fMRI

The *R* platform has important benefits for fMRI analyses because of its availability and functionality. *R* is free and open source, so licensing costs for research are not prohibitive and any researcher is able to install it easily. Because of this, sharing code to validate methods and reproduce findings is quite simple. *R* contains thousands of packages that can perform cutting-edge statistical and machine learning techniques; analyses and hypothesis are not limited by the available models. *R* allows the user direct contact with their data, with routines for fMRI that can efficiently extract single timeseries, volumes, or planes. This is of particular value because fMRI scans are encoded in specialized formats (ANALYZE or NIfTI) that are otherwise unaccessible. The ability to access directly the data combined with the high-level statistical methods available within the general *R* framework allows the user to construct his own methods unique to his hypothesis.

Finally, *R* contains packages to implement specifically fMRI analysis. There are routines pre-built into *R* for fMRI that can perform methods such as mixed effects analysis **3dMEMA** (Chen et al., 2010, 2012) to estimate the effect, which is implemented indirectly in *R* by sourcing through AFNI (http://afni.nimh.nih.gov/sscc/gangc/MEMA.html). The bread and butter of fMRI analysis, the general linear models (GLM) can be implemented in the package **fmri** (Polzehl and Tabelow, 2007), Bayesian multi-level modeling analysis in **cudaBayesreg** (Ferreira da Silva, 2011) and Granger Causality and structural equation modeling in **FIAR** (Roelstraete and Rosseel, 2011). Functional connectivity analysis also can be performed using the package **brainwaver**, where ROIs are analyzed for connectivity using wavelet analysis, and connections are trimmed with a hypothesis test (Achard et al., 2006). We refer the reader to a presentation at the use-R! 2010 conference for a description of packages and options in *R* for fMRI analyses, at http://www.r-project.org/conferences/useR-2010/slides/Chen+Saad+Cox.pdf. Some packages require installing the most recent versions of gfortran and tcltk available for MacOS at http://cran.r-project.org/bin/macosx/tools/.

## 3. Methods

**Figure 1:**
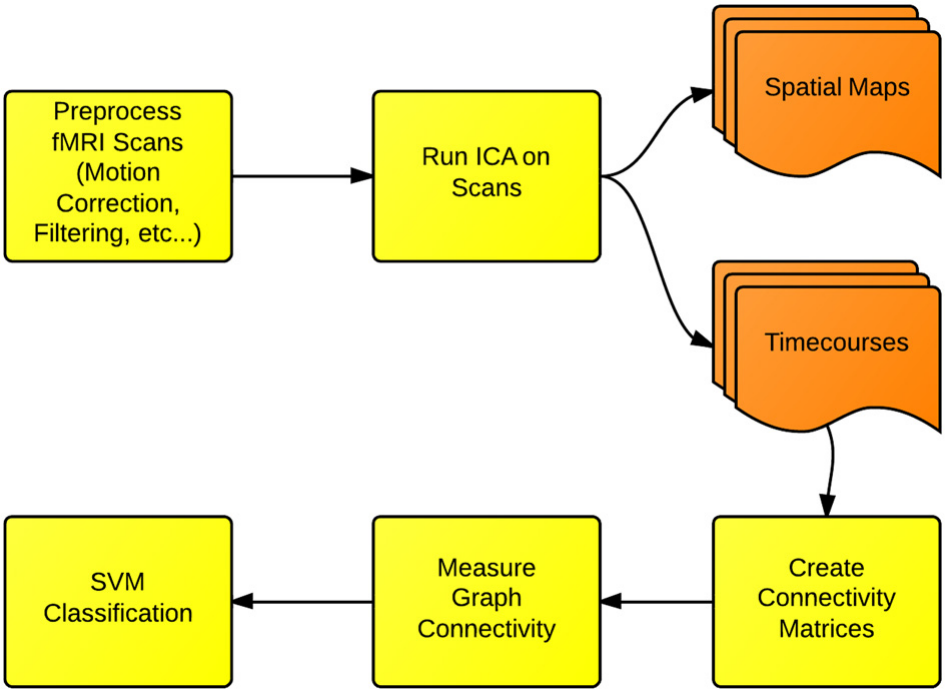
**Functional network connectivity (FNC) and classification: the first step in FNC is to define the scale of connectivity to observe.** In this case, we use whole-brain networks obtained from ICA, but this analysis also can be implemented on the region-of-interest or the voxel scale. The connectivity is defined and measured to identify differences between either groups or conditions.

In this section, we cover the specific methods used for FNC and fMRI classification presented in this paper. We first use the ICA dimension-reduction technique to decompose each scan into a set of spatial brain networks being modulated over time by associated timecourses. We then create functional connectivity matrices, by measuring the longitudinal correlations of the timecourses for each network. Next, each matrix is converted into a graph structure, and the connectivity properties of each graph are measured. Finally, these connectivity properties are used as features for an SVM classifier. We additionally use a t-test to evaluate whether the small-world connectivity of ICA networks is different between Schizophrenia patients and healthy controls. A flowchart depicting this process is shown in Figure 1.

### 3.1. ICA for fMRI

As fMRI is composed of recordings that are highly-redundant in both space and time, it is desirable to extract meaningful features prior to classification. This serves two purposes. Firstly, it lowers the noise by essentially tossing out signals that have no commonality with other signals. This is based on the assumption that noise is independent across observations; a signal seen only in a single location is more likely to be noise than a signal observed consistently throughout the brain. Secondly, reducing the scan to a manageable number of consistent signals reduces the tendency of overfitting in the classification process. The classification complexity is a function of the number of dimensions (features).

Although there are many methods of extracting common signals across the brain, ICA in particular has gained popularity in fMRI. It can isolate networks corresponding to neurological activity, as well as motion artifacts, where signals that operate most strongly on the peripheral regions along the scalp are taken to be motion. ICA has been validated through bootstrapping and clustering methods, identifying components that exist across subjects and scans that correspond to functionally identifiable brain networks (McKeown et al., 2003; Anderson et al., 2011). In this implementation we run ICA within subjects, rather than implementing a group-ICA which would have identified common networks across all subjects. This is based upon the hypothesis that there are a different set of networks operating within Schizophrenia, and assuming that the same exact networks operate within both patient groups would dampen any between-group differences.

**Figure 2:**
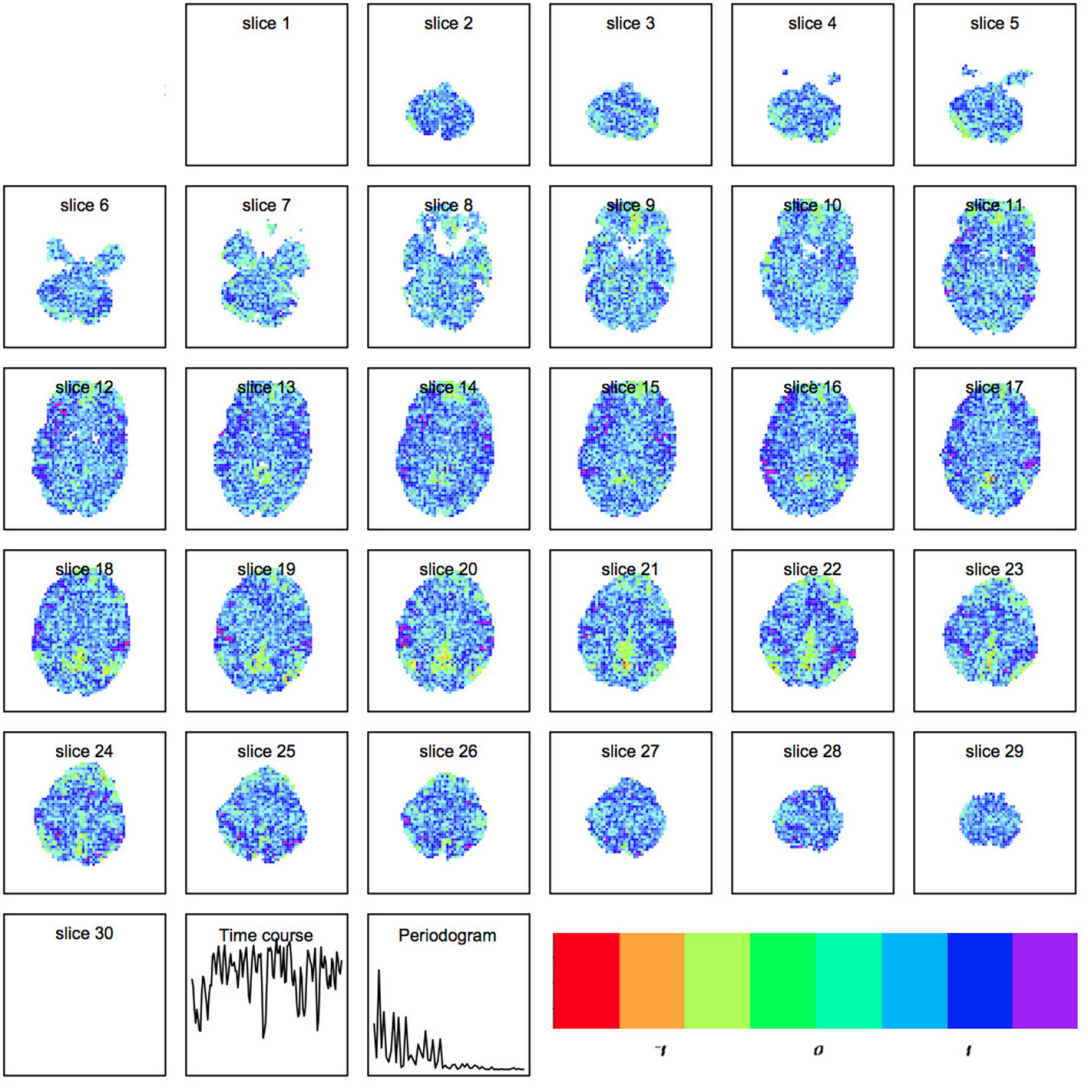
**Spatial map produced by independent components analysis within R.** Each component is a set of spatially weighted regions modulated by the time course. The total longitudinal contribution of a component to the activity observed is the spatial map multiplied by the timecourse.

Under the hypothesis that the activity of the brain is constructed of anatomical networks acting together to produce meaningful psychobehavioral cognitive states, the aggregate activity is decomposed into subcomponents in ICA. Prior to this, space is “unrolled” where the four dimensional scan (3 dimensions of space, 1 of time) are transformed into a matrix of dimension space by time, so that a scan array of dimension (*X*, *Y*, *Z*, *T*) would become a matrix of dimension (*T*, X*Y*Z). An fMRI scan of time length *T* and spatial dimension *S* and can be expressed as a linear combination of *M* < *T* components and the corresponding timeseries:

$$X_{ts}=\sum_{\mu =1}^{M}A_{t\mu }C_{\mu s}$$

where *X*_*ts*_ represents the raw scan intensity at timepoint *t* ≤ *T* and spatial location *s* ≤ *S*, *A*_*t*μ_ is the amplitude of component μ at time *t*, and *C*_μ*s*_ is the spatial magnitude for component μ at spatial location *s*. An example of a spatial map output by *R* is shown in Figure 2.

The components *c* are estimated to be statistically independent as possible by solving instead the inverse problem via the FAST-ICA algorithm. To estimate the signal sources *c* in *x* = *Ac*, the inverse problem of *y* = *w*′*x* is solved where *w* is a row of *A*^−1^, or the inverse of the mixing matrix. Then *y* = *w*′*x* ⇒ *y* = *w*′*Ac*. Substituting *z* = *A*′*w*, *y* = *z*′*c*. *W* is optimized such that *y* = *w*′*x* = *z*′*c* is as non-Gaussian as possible, leading to even *less* Gaussian sources *c* because of the Central Limit Theorem. Maximizing the kurtosis, minimizing the entropy, and maximizing the negentropy over *w* are all methods of finding the *least* Gaussian *y* = *w*′ *x* = *z*′*c*.

$$\begin{array}{l} Negentropy=J(y)=H(y_{Gauss})-H(y)\\ \;H(y)=-\sum_{i}P(y=a_{i})log[P(y=a_{i})] \end{array}$$

In the continuous case this becomes

H(y)=−∫f(y)log(f(y))dy

By default *R* and FSL use FAST-ICA. The default parameter setting in *R* is for parallel extraction, and also includes temporal normalization, 1000 maximum iterations for the algorithm using negentropy: $G(u)=\frac{1}{\alpha }log[cosh(\alpha u)]$ where α ∈ [1, 2] is the constant used for the negentropy approximation. An example of a ICA spatial map is shown in Figure 2.

We implement here spatial ICA the only option in FSL, which sought statistical independence of the spatial maps. We alternatively could have implemented temporal ICA, which would have maximized independence of the time-courses. A presentation of this using the **AnalyzeFMRI**, and a demonstration of how to implement temporal ICA within R using the **AnalyzeFMRI** is provided in (Bordier et al., 2011). We allowed the number of components to be determined within the data following (Allen et al., 2011).

### 3.2. Creating functional connectivity matrices

**Figure 3:**
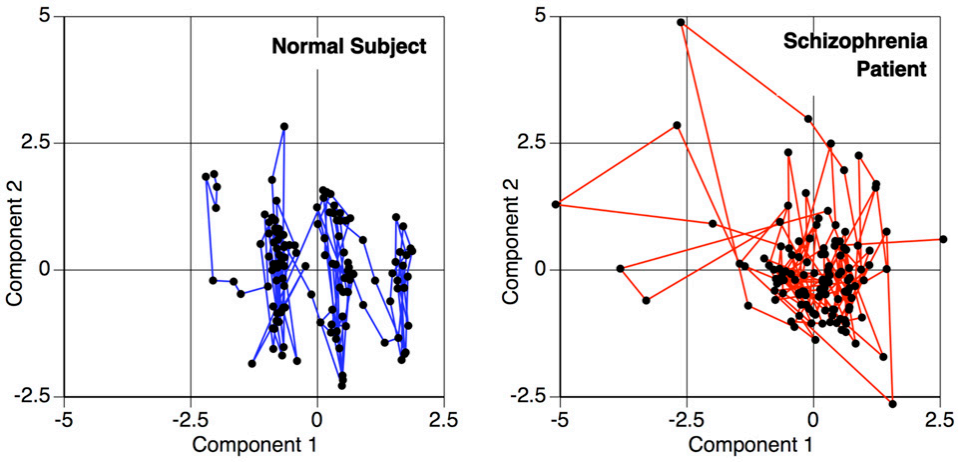
**Temporal activity plot of two primary components within a subject, depicting the relationship between two components over time.** This phase space transition between pairs of components are measured for the functional connectivity analysis, to calculate the similarity of the components' behavior.

A temporal interaction plot for a schizophrenia patient and a normal control is shown in Figure 3, showing the joint longitudinal activity by two components within each subject, (*A*_μ_1__, *A*_μ_2__). Since graphical interpretation is subjective, a fixed measure of this joint activity is established by computing a correlation-based distance metric. The distance function is a transformation of the maximal absolute cross-correlation between two timeseries. This computation is done for each possible pair of components within a subject, thus transforming the original fMRI scan into a matrix. This is a measure of the functional connectivity between components for a given subject, but is only one of many possible metrics that can be changed by the end user within this tutorial. This is but one example where *R* allows the user to change the methods according to the hypothesis and data being evaluated.

The cross-correlation function (CCF) between these timeseries is calculated over a range of temporal lags. We subtract the maximal absolute cross-correlation from 1 to create a pseudo distance measure, *d*(*A*_μ_*i*__, *A*_μ_*j*__), given by

$$d(A_{\mu_{i}},A_{\mu_{j}})=1-max[|CCF(A_{\mu_{i}},A_{\mu_{j}},l)|]$$

where

(1) $$CCF(A_{\mu_{i}},A_{\mu_{j}},l)=\frac{E[(a_{\mu_{i},t+l}-\bar{A_{\mu_{i}}})(a_{\mu_{j},t}-\bar{A_{\mu_{j}}})]}{\sqrt{E[{\left(a_{\mu_{i},t}-\bar{A_{\mu_{i}}}\right)}^{2}]E[{\left(a_{\mu_{j},t}-\bar{A_{\mu_{j}}}\right)}^{2}]}}$$

where *l* is the time lag separating the two timeseries *A*_μ_*i*__ and *A*_μ_*j*__, and $\bar{A_{\mu_{i}}}$ is the mean of the entire timeseries *A*_μ_*i*__ = (*a*_μ_*i*_, 1_, *a*_μ_*i*_, 2_, …, *a*_μ_*i*_, *T*_). The timeseries are calculated at lags ranging from 0 to 3 points (6 seconds), as higher lags results in fewer time points to calculate the correlation and a more noisy estimate, and also lacks biological plausibility given our current understanding of neurological coupling. Within *R*, the lag parameter is specified using lag.max.

**Figure 4:**
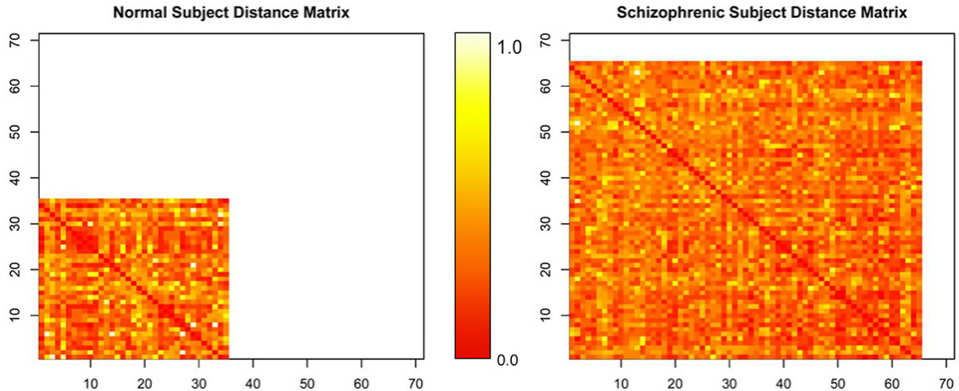
**Normalized distance matrices of two subjects, where rows and columns correspond to components within a subject and the intensity represents the functional connectivity between those components**.

The matrices by themselves are uninterpretable, since they are merely representations of a set of connected objects. An example of this is shown in Figure 4. Moreover, the rows and columns of these matrices, representing unique independent components within subjects, are themselves not comparable across subjects. Our ultimate goal is to measure this connectivity; not only how closely connected they are, but also how it changes with respect to patient diagnosis. For example, do all networks interact with all other networks? Are there subgraphs that are fragmented from the original graph? Does the number of steps to travel among nodes differ? Are some graphs more densely connected than others? To answer these questions, we must convert the connectivity matrices to graph objects, so we can use *R* packages designed purposefully for graph connectivity analysis.

### 3.3. Graph creation and measurement

Each matrix represents a structure of completely connected points on a high-dimensional manifold, where each point is an independent component and the distance between two points measures the similarity of their temporal activity. Every point is linked to every other point regardless of similarity. To create graphs out of the connectivity matrices, we prune weak connections among points and then embed the simplified structure into a lower-dimensional space using the ISOMAP procedure (Tenenbaum et al., 2009). Then, we measure the graph-theoretic connectivity to summarize the connections between resting-state networks.

#### 3.3.1. Graph creation

**Figure 5:**
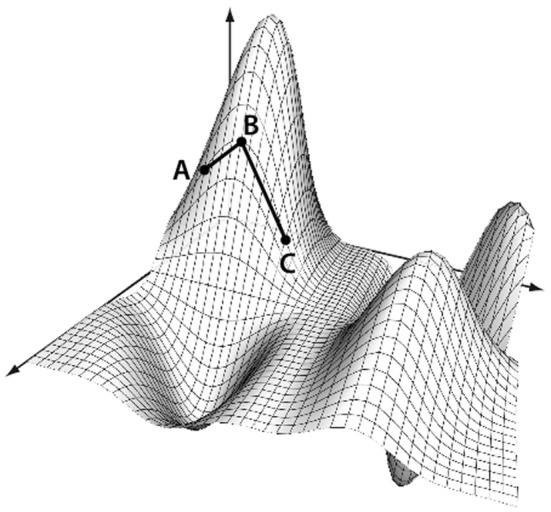
**Geodesic distance calculation.** The distance between A and C is calculated as the manifold path distance from A to B to C, instead of the direct path from A to C. This eliminates the assumption that the points occupy a linear space when using a Euclidean distance.

Conceptually, any set of points contained in a distance matrix of dimension *d* can be embedded into a space of dimension *d* − 1 without any information loss (preserving all the distances between points). Usually such a transformation assumes the space on which the points lie is linear. This, however, may not be the case. Consider if you were trying to measure the distance from Sacramento, California to Shanghai, China, using only the (x,y,z) grid coordinates of each city. The linear distance between the cities, while calculable, would assume that the correct path from Sacramento to Shanghai went through the core of the earth. Instead, a more reasonable way to measure the distance would be to travel along the flight-paths, from Sacramento to Los Angeles, Los Angeles to Tokyo, and finally from Tokyo to Shanghai. This method of measuring distance is known as the geodesic distance, or path-distance among points. It assumes that travel among distant points usually requires routing through intermediate nodes, as shown in Figure 5. It is this concept we will now use to sever weak connections and create graphs out of the matrices.

**Figure 6:**
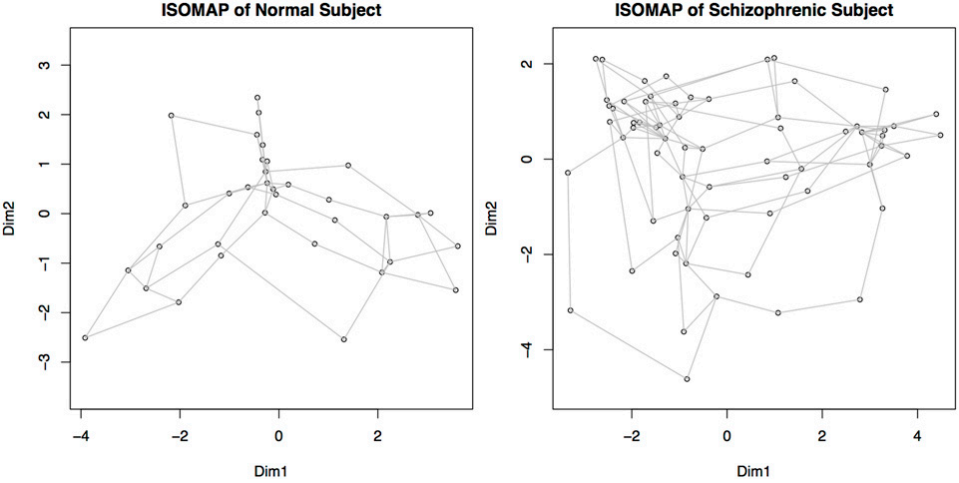
**Graphs representing connectivity of two subjects, obtained by converting the distance matrices for each subject into a structure where each node represents a component, and the distance between nodes represents the connectivity or similarity of their behaviors.** Nodes close together demonstrate a higher functional connectivity measure. This map is obtained by recalculating the connectivity matrices using geodesic distances, and then embedding the points in a two dimensional space for plotting. Dim 1 and Dim 2 represent the weightings on the two primary dimensions, similar to multi-dimensional scaling.

We transform each matrix into a graph structure using an initial geodesic distance calculation implemented in the function isomap in the library **vegan**. Weak ties among points are then severed; points can be connected if they are within a certain distance, ϵ, of each other (|*x* − *y*| ≤ ϵ), or they can be connected if they are within a set of *k*-nearest neighbors. The distances are recomputed after pruning, where the distance between connected points is the same as it was originally, but the new distance between unconnected points is computed as the shortest path through intermediary connecting nodes. Combined with multidimensional scaling to obtain a coordinate system for embedding, this procedure is called ISOMAP (Tenenbaum et al., 2009). An example of such a graph created by a geodesic distance transformation and a multidimensional scaling embedding is shown in Figure 6.

The two definitions of connectivity (nearest-neighbor vs. ϵ-distance) can lead to different results; establishing connectivity by ϵ-distance, also called edge density (sparsity), may lead to the graph becoming fragmented, with some portions of the graph having no connections, direct or indirect, to other subgraphs. This would be caused by some point(s) being too distant to others to maintain a connection with the main graph. This is an instance where the *a priori* knowledge about the disease may inform the parameter choices of the methods. In diseases such as Schizophrenia or autism, a hypothesis of disconnectivity may be tested directly by computing, for example, the fragmentation rate of the patients versus controls. By allowing the user to choose these parameters within *R*, specific theories of neuropathology may be tested.

We threshold points as being connected using a modified nearest neighbor method. We select the k-nearest neighbors of all nodes to be connected by defining k as 10% of total components for that subject, or 2, whichever is greater. This enforces the graph be completely connected, unlike the edge density method. We select this parameter choice because we are using weighted graphs; edge density methods typically binarize the adjacency matrix by assigning weights above a given threshold a unit value, and a zero value to all below such as in Rubinov and Sporns (2010). Since we are using weighted graphs, we allow sufficiently “close” points to retain their given weights, and prune all other points which are not sufficiently close. This parameter could be investigated further, but because we are using these metrics for performing classification then optimizing the adjacency pruning method would lead to biased estimates of the accuracy.

#### 3.3.2. Graph measurement

At this point, each brain scan has been transformed into a graphical structure, where each node represents a brain network and the connectivity between nodes represents the similarity in the activity of these networks. Each graph can then be summarized by its connectivity properties. There are many such measurements available within *R* within the package **igraph**. A tutorial by Gabor Csardi on Network Analysis with the package **igraph** is at http://igraph.sourceforge.net/igraphbook/. A description of network measures of brain connectivity is available at Rubinov and Sporns (2010), which describes in detail the graph-theoretic measures discussed only briefly here, and additionally describes other connectivity measures such as modularity. An additional connectivity measure which we used in a previous study to discriminate between Schizophrenia patients and healthy controls (Anderson et al., 2010) is the “eigenvector centrality,” which can be computed here using the command eigen(d)$values.

Creating graphs out of each matrix using a non-linear distance metric such as the geodesic distance not only allows for a more efficient low-dimensional projection of the matrix, but also encourages the graph to be connected more efficiently by trimming poor connections while maintaining stronger ones. This fragmentation allows us to determine how many strong connections are within the subject, how many subnetworks (subgraphs) exist, what the sizes of these subnetworks are, and how efficiently the points are connected overall. These properties, all interrelated, give quantitative measurements of the connectivity that can be used to fingerprint the networking differences associated with different disorders. These individual metrics can be compared directly between groups if multiple comparisons are adjusted for.

Some of these available measures within **igraph** are:

- Average path length: average path length between all connected vertices.
- Clique number: number of elements in the largest subgraph.
- Graph density: ratio of the number of edges to the number of possible edges.
- Edge connectivity (also called graph adhesion): minimum number of edges needed to obtain a graph which is not strongly connected.
- Median closeness: median number of steps required to access every other vertex from a given vertex.
- Median Degree: median number of edges incident to a vertex, with loops being counted twice.
- Vertex Count: number of Vertices in the graph.
- Edge count: number of Edges in the graph.
- Maximum degree: maximum number of edges incident to a vertex, with loops being counted twice.
- Transitivity: probability that two vertices are connected. This is also called the *clustering coefficient*.

We use two of these measures to compute the *small-world* property generated by the Erdős-Renyi game (Erdős and Rényi, 1961). Alternative methods of computing this are presented by Rubinov and Sporns (2010). The small-world measure σ is computed as

$$\sigma =\frac{\gamma }{\lambda }$$

where γ is the ratio of the clustering coefficient of the real network to the mean of the clustering coefficient of *n* random networks with an equivalent number of edges and weights as the real (data-derived) network but randomly rewired. λ is the similar to γ but uses characteristic path length. The variable *n* is usually somewhere between 500 and 5000. Typically, biological networks have:

- γ >> 1, i.e., greater local clustering than a random network
- λ ≈ 1, i.e., similar characteristic path length to a random network

Any network with σ > >1 is considered to be “small world” (Humphries and Gurney, 2008).

### 3.4. Classification using SVM

We have transformed each subject's fMRI scan into a graph, where the nodes of the graph represent functional networks and the distances between nodes represents the similarity of the activity of the nodes. We have measured the connectivity of these graphs, or the FNC. We now wish to use these connectivity features for classification. *R* has an immense number of libraries available for classification. Packages are continually being added that implement new machine learning algorithms, and packages for specific algorithms can be found at http://www.rseek.org. In this paper we use the basic SVM algorithm, included in the package **e1071**.

The SVM algorithm attempts to find a hyperplane that best separates different classes, using only the points contained in the margin (or region of overlap.) For a set of points (*x*_*i*_, *y*_*i*_) where (*x*_*i*_) ∈ *R*^*n*^ is the set of graph measurements for the graph *G*_*i*_ corresponding to subject *i*, a member of class *y*_*i*_ ∈ (−1, 1) (patient or control), SVM will learn the hyperplane which best divides the classes (−1, 1). If a hyperplane is modeled by *w* · *x*_*i*_ − *b* where *w* are the vectors normal to the hyperplanes, the parallel hyperplanes separating the observations can be defined by *w* · *x*_*i*_ − *b* ≥ 1 for *y*_*i*_ = 1 and *w* · *x*_*i*_ − *b* ≤ −1 for *y*_*i*_ = −1. The optimization problem becomes to maximize the distance between planes, $\frac{2}{\left\|w\right\|}$, such that *y*_*i*_(*w* · *x*_*i*_ − *b*) ≥ 1

Using the graph properties (path length, clique number, etc…) as features, we can then perform classification between schizophrenia patients and healthy controls.

## 4. Applications

### 4.1. Preprocessing and component extraction in FSL

**Figure 7:**
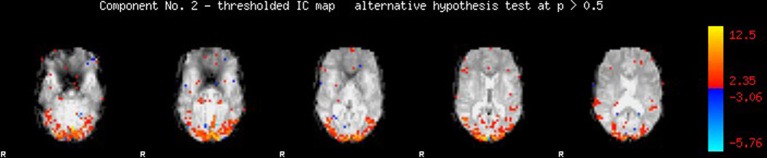
**Spatial map produced by independent components analysis in FSL**.

Our first step was to perform motion correction and skull-stripping on the fMRI data, and to run ICA within each scan to extract the networks of interest. We specifically use automatic component estimation in FSL because our previous research has suggested differences in the number of independent components for Schizophrenia patients and healthy controls. An example of an ICA map produced by FSL is shown in Figure 7.

Assuming the COBRE data has been downloaded and installed, then we can run FSL from the command line to process all scans, which includes motion correction, skull-stripping, smoothing with a 6 mm filter, high-filtering at 100 Hz, and finally running Melodic with automatic component estimation within each subject. This script is tailored to the COBRE data, and requires that the variable STUDY_DIR be changed for the users' specific path. Following this script the *melodic_mix* files containing the patients' ICA timecourses will all be located in the folder ./COBRE/COBRE_MELODIC. To create a similar script for a new dataset, one can simply run Melodic from the GUI, and copy the command-line input from the *log* file.


#!/bin/sh
# Subject directories (Change Me)
STUDY_DIR=“/u/home/of/my/COBRE”;
#############
# CONSTANTS #
#############
subjects=“0040000  0040005  0040010  0040015  0040020  0040025  0040030  0040035  0040040
0040045  0040050  0040055  0040060  0040065  0040070  0040075  0040080  0040085  0040090
0040095   0040100  0040105  0040110  0040115  0040120  0040125  0040130  0040135  0040140
0040145  0040001  0040006  0040011  0040016  0040021  0040026  0040031  0040036  0040041
0040046   0040051  0040056  0040061  0040066  0040071  0040076  0040081  0040086  0040091
0040096   0040101  0040106  0040111  0040116  0040121  0040126  0040131  0040136  0040141
0040146  0040002  0040007  0040012  0040017  0040022  0040027  0040032  0040037  0040042
0040047   0040052  0040057  0040062  0040067  0040072  0040077  0040082  0040087  0040092
0040097   0040102  0040107  0040112  0040117  0040122  0040127  0040132  0040137  0040142
0040147  0040003  0040008  0040013  0040018  0040023  0040028  0040033  0040038  0040043
0040048   0040053  0040058  0040063  0040068  0040073  0040078  0040083  0040088  0040093
0040098   0040103  0040108  0040113  0040118  0040123  0040128  0040133  0040138  0040143
0040004   0040009  0040014  0040019  0040024  0040029  0040034  0040039  0040044  0040049
0040054   0040059  0040064  0040069  0040074  0040079  0040084  0040089  0040094  0040099
0040104   0040109  0040114  0040119  0040124  0040129  0040134  0040139  0040144”;

umask 0002;
######################
# PROCESSING COMMANDS #
######################

# Change to STUDY_DIR
cd $STUDY_DIR;
mkdir COBRE_MELODIC

# Loop through subjects
for i in $subjects; do
         if [ ! -f “$STUDY_DIR/COBRE_MELODIC/${i}_melodic_mix_new” ]; then
         cd $STUDY_DIR/${i}/session_1/rest_1
         rm -r *.ica*
         rm rest_mcf*
             rm prefiltered*
             rm filtered*
             mcflirt -in rest.nii.gz -out prefiltered_func_data_mcf -mats -rmsrel -rmsabs
             fslmaths prefiltered_func_data_mcf -Tmean mean_func
             bet2 mean_func mask -f 0.3 -n -m; immv mask_mask mask
             fslmaths prefiltered_func_data_mcf -mas mask prefiltered_func_data_bet
             fslstats prefiltered_func_data_bet -p 2 -p 98
             fslmaths prefiltered_func_data_bet -thr 100.8095459 -Tmin -bin mask -odt char
             fslstats prefiltered_func_data_mcf -k mask -p 50
             fslmaths mask -dilF mask
             fslmaths prefiltered_func_data_mcf -mas mask prefiltered_func_data_thresh
             fslmaths prefiltered_func_data_thresh -Tmean mean_func
             susan prefiltered_func_data_thresh 614.340225 2.12314225053 3 1 1
                         mean_func 614.340225 prefiltered_func_data_smooth
             fslmaths prefiltered_func_data_smooth -mas mask prefiltered_func_data_smooth
             fslmaths prefiltered_func_data_smooth -mul 12.2082189881 prefiltered_func_
                         data_intnorm
             fslmaths prefiltered_func_data_intnorm -bptf 25.0 -1 prefiltered_func_
                         data_tempfilt
             fslmaths prefiltered_func_data_tempfilt filtered_func_data
             fslmaths filtered_func_data -Tmean mean_func
             melodic -i filtered_func_data ––nobet ––bgthreshold=3 ––tr=2.0000000000 -d 0
                         ––mmthresh=0.5
             cp filtered_func_data.ica/melodic_mix
                         $STUDY_DIR/COBRE_MELODIC/${i}_melodic_mix_new
fi
done  #END “Loop through subjects...”
echo “Processing complete.”;


### 4.2. Graph creation and measurement in R

#### 4.2.1. Computing functional network connectivity using lagged correlations

FNC requires breaking down the original temporal scans into a series of units modulated by time-series, where the correlations of the timeseries determines their similarity. We begin by calling the necessary libraries for this analysis:


> library(igraph)
> library(vegan)
> library(e1071)


For the COBRE data we can create the list of filenames using:


> setwd(“/path/to/my/directory”)
> filenames <-
             dir (pattern=“melodic_mix_new”)


Otherwise, we can read in the filenames from a text document using the scan() command.

With FSL the number of ICA components was determined uniquely for each subject. We first determine the number of components within each file to store within the vector, **s**.


> num_subjects <- length(filenames)
> s <- c(rep(0,num_subjects))
> for (i in 1:num_subjects)
{     s[i] <- dim((read.table(as.character
               (filenames[i]))))[2]  }


We next read in each melodic_mix file and use these to create a distance matrix. We define the distance matrix as the maximal normed cross-correlation for a lag distance of 3. The distance between the timeseries for each component is calculated and stored in data_array_distance. The mapply function is used to apply the distance function to all elements in the upper-triangular part of the distance matrix, instead of using a nested for loop to calculate each item individually.


> data_array_distance <- array(NA, c(num_subjects, max(s), max(s)))
> for (i in 1:num_subjects)
{        ##Read in ICA results
        temp <- as.matrix(read.table(as.character(filenames[i])))
        for(j in 1:s[i])
        {        for(k in j:s[i])
                 {        data_array_distance[i,k,j] <- 1-max(abs(ccf(temp[,j], temp[,k],
                                          plot = FALSE, lag.max = 3)$acf))
                         data_array_distance[i,j,k] <- data_array_distance[i,k,j] }}  
        diag(data_array_distance[i,,]) <- 0    }


At this point the fMRI scans of each subject have been converted: first by decomposing them into independent components, and then creating a functional connectivity matrix measuring the temporal connectivity among components within each subject. Every *melodic_mix* file has been converted into a functional connectivity distance matrix.

#### 4.2.2. Graph creation and analysis

Next, we transform each matrix into a graph structure and measure the connectivity properties of each graphs. We first use the ISOMAP embedding algorithm to compute the distances among elements using the geodesic framework, and prune weak connections with package **vegan**. We then create a graph structure whose connectivity can be measured using functions in **igraph**. Because the data type output in **vegan** is different than the type needed for **igraph**, we create an internal conversion function named makegraph. This graphical structure uses weighted edges in a dissimilarity matrix, where ‘0’ indicates that two points are not connected. Because of this, we use the inverse of the distance to define the weights between two vertices when the ISOMAP algorithm has computed they are connected.


> makegraph <- function(my_iso)
{               ##dim is dimension of matrix
                my_dist <- as.matrix(dist(my_iso
                                               $points[]))
                k <- dim(my_dist)[1]
                my_net <- matrix(0, nrow = k,
                                              ncol = k)
                which.rows <- my_iso$net[,1]
                which.cols <- my_iso$net[,2]
                for(j in 1:length(which.rows))
                {      my_net[which.rows[j],
                         which.cols[j]]
                         <- 1/my_dist[which.rows[j],
                         which.cols[j]]
                       my_net[which.cols[j],
                         which.rows[j]]
                         <- 1/my_dist[which.cols[j],
                         which.rows[j]]
                                }
                my_net        }


We next analyze the properties of these graphs. We create a function to calculate the coefficients γ and λ of a random graph. This is used to compute the *small worldness* of the actual graph proposed by the data. We call this function smallworld; it takes in 2 parameters: *n* = the number of vertices, and *m* = the number of edges. The value γ is a transitivity measure, of the probability that adjacent vertices are connected. This is sometimes called the *clustering coefficient*. This function is called later to compute the values γ and λ later for the randomly reconnected graph, and is averaged across 5000 random graphs. These values are used to form the ratio to calculate σ, the small-worldness measure.


> smallworld <- function(n,m)
{  smallworld <- matrix(nrow=5000,ncol=2)
    for(k in 1:5000)
    {   g  <-  erdos.renyi.game
                         (n,m, type=“gnm”)
        smallworld[k,1] <- transitivity(g)
        smallworld[k,2] <- average.path.
                                length(g) }
    colMeans(smallworld)    }


We next run our geodesic-distance pruning procedure (ISOMAP) within **vegan**, convert the data structure using our function makegraph, and then measure the connectivity using **igraph**. We compute separately the small-world measure, σ, which is a vector output, by the routine called my_small_worldness. We threshold points as being connected using the k-nearest neighbors method.


> my_small_worldness <- matrix(NA, nrow = num_subjects, ncol = 1)
> my_feature_matrix <- matrix(NA, nrow = num_subjects, ncol = 12)
> randomsmallstore <- matrix(NA, nrow = num_subjects, ncol = 2)
> for(i in 1:num_subjects)
{ d <- matrix(data_array_distance[i,1:s[i],1:s[i]], nrow = s[i])  
   my_iso <- isomap(d[1:s[i],1:s[i]],axes=3, k=max(floor(s[i]/10),2), ndim = 15,
       fragmentedOK=TRUE)
   my_net <- makegraph(my_iso)
   d2 <- graph.adjacency(my_net )  
   transitivity(d2)
   n=vcount(d2)
   m = ecount(d2)
   randomsmall <- smallworld(n,m)
   sigma <- (transitivity(d2)/randomsmall[1])/(average.path.length(d2)/randomsmall[2])
   randomsmallstore[i,] <- c(randomsmall)
   my_small_worldness[i,] <- sigma  
   my_net <- makegraph(my_iso)
   d2 <- graph.adjacency(my_net, weighted = TRUE )
   my_feature_matrix[i,] <-c(average.path.length(d2),clique.number(d2),graph.density(d2),
       edge.connectivity(d2),median(closeness(d2)),median(graph.coreness(d2)),
       max(degree(d2)),median(degree(d2)),min(degree(d2)),vcount(d2),ecount(d2),
       transitivity(d2))  }
> my_feature_matrix <- cbind(my_feature_matrix,my_small_worldness)


Finally, we label the columns.


> colnames(my_feature_matrix) <- c(“Average Path Length”, “Clique Number”,“Graph Density”,
    “Edge Connectivity”, “Median Closeness”, “Median Graph Coreness”,“Max Degree”,
    “Median Degree”,   “Min Degree”, “Vertex Count”, “Edge Count”,“Transitivity”,
    “Small Worldness”)


We began with functional connectivity matrices, turned each matrix into a graph, and measured the connectivity of each graph. We used a total of 13 connectivity measures, include the small-world calculations. The feature vectors collectively form a feature matrix that will be used for classification in the following section.

### 4.3. SVM classification in R

In this section we demonstrate SVM Classification using the package **e1071** that contains an interface to the libsvm *C++* package by Chih-Chung Chang and Chih-Jen Lin. The *R* vignette (http://cran.r-project.org/web/packages/e1071/vignettes/svmdoc.pdf) details the functionality of this package, which includes many other classification routines besides SVM. The SVM method within this package has an optional benefit of cross-validation, which simplifies coding dramatically by implementing the training and testing steps within a single function call. In the following code we demonstrate classification of our feature vector using 10-fold cross-validation, but this is an adjustable parameter. There are many options within the svm() method that can be specified such as kernel choices, but we use the default parameters here (“radial basis function”) for the sake of conciseness and clarity.

We create a vector my_cat with the response variables, in the case the patient diagnosis of each scan. This can alternatively be read in using the function read.table(). Because within the COBRE data two patients were disenrolled, we exclude those patients from this analysis. As R read in the list of files alphabetically and the COBRE demographic spreadsheet has patients entered in order of age, we reorganize the file formats to ensure that our patient labels read in from the spreadsheet match up with the data matrix already created.


> cobre <- read.csv
           (“COBRE_phenotypic_data.csv”)
> cobre <- cobre[order(cobre[,1]),]
> my_cat <- cobre[,5]
> my_data_matrix <- my_feature_matrix
                   [my_cat!=“Disenrolled”,]
> my_cat <- my_cat[my_cat!=“Disenrolled”]
> my_data_matrix <- cbind
                  (my_cat,my_data_matrix)


Finally, the library is loaded and the model is trained and tested using 10-fold cross-validation.


> my_svm <- svm(as.factor(my_cat).,
  data = as.data.frame(my_data_matrix),
  cross=10)
> pred <- fitted(my_svm)


The structure my_svm contains many details of the model. We can see the average cross-validation accuracy within each *k*^th^-fold using my_svm$tot.accuracy.

### 4.4. Hypothesis testing

Alternatively, we can test for between-group differences using formal hypothesis testing. For example, if we wished to test the individual metrics between patients and controls, we could do so using:


> for(i in 2:14)
{  print(t.test(na.omit(my_data_matrix
                    [my_cat==“Patient”,i]),
        na.omit(my_data_matrix[my_cat=
                     =“Control”,i]))) }


All the computed graph-connectivity measures are correlated; for example, a graph with a low *median closeness* measure would imply that there is a short distance between two vertices, thus increasing the *transitivity*. Because of this, to compare the 13 measures we would have to adjust for multiple comparisons. Using a Bonferroni correction, only *p*-values below 0.05/13=0.0038 would be considered significant.

## 5. Results and discussion

We briefly present here the results of this analysis.

Patients had a significantly lower clustering coefficient than healthy controls (*p*< 0.05, corrected). Lower clustering implies networks are less likely to be connected to each other in Schizophrenia, indicating that the networks are themselves less synchronized and more independent of each other. Patients had lower small-world measures of connectivity than healthy controls, although both groups exhibited small-world connectivity among independent components. This difference was not statistically significant when using unweighted graphs, but was statistically significant when using weighted graphs.

Using just the scripts provided here, our 10-fold cross-validation accuracy is 65%, compared to a chance accuracy of 50.7%. There are quite a number of things we could do to improve this accuracy, which we omitted intentionally here because they are outside the scope of this tutorial. We performed no quality-control on this data to exclude scans with excessive motion or scanner artifacts. We also took no measures to identify and exclude ICA networks that were related to motion, scanner noise, or physiological artifacts. We did not use any of the demographic information (patient gender, age, etc…) which would likely have improved accuracy, both by controlling for functional brain changes and also by controlling for sampling variation. For example, in this sample males were more likely to be Schizophrenia patients than females, so knowing this information would have permitted classification based upon this information, which is parallel to the actual functional connectivity analysis. Finally, we implemented only the basic SVM algorithm without any parameter tuning, and similarly did not optimize the definition of “connectivity” among points. Connectivity definitions have been shown previously to affect the final results, with different thresholds for connectivity having significant effects on the final graph-theoretic measurements (Toppi et al., 2012). Given the simplicity of our methods, it is perhaps somewhat remarkable that we were able to achieve the classification accuracy realized here and significant small-world differences between patients and controls.

## 6. Conclusion

Collectively, we have provided methods to determine whether functional connectivity differences exist between groups, and to demonstrate that the resting-state functional connectivity differences in schizophrenia can be useful for automated patient diagnosis. Functional connectivity measures can be used to discriminate between patients and controls, and schizophrenia patients show lowered clustering of networks than healthy controls, indicating that networks within Schizophrenia are more disconnected.

The analysis outlined here is intended to be adjusted and altered by the end user, even those who aren't regular users of *R*. The user has flexibility in altering parameters such as distance metrics, classification machines, and feature selection choices. For example, another method of implementing functional connectivity is through Granger causality among ROIs such as in Sato et al. (2010), whereas this presentation implements functional connectivity through correlations among functional networks determined by ICA. Other distance metrics could have been used, which would be optimal given the recent finding that using correlation metrics to compute distance automatically leads to non-random graphical structures (Zalesky et al., 2012). We performed SVM classification in *R*, a “black-box” model which ironically is remarkably simple to implement with a single function call to both train and test the model using cross-validation. Because *R* is an established package in the statistics research community, many newer machine learning procedures can easily be implemented to compare with more established classification machines.

This analysis and tutorial is not without limitations; primarily, we took no steps to identify and discard artifacts in the ICA data, which almost certainly would have increased the classification “accuracy” we obtained. This omission was intentional given the intentions of this tutorial; manually identifying artifacts within ICA is outside the scope of this manuscript, and other tutorials to perform to identify fMRI artifacts and clean data further are available elsewhere. We wish here to illustrate how functional connectivity can be measured in a graph-theoretic approach, and to provide a working framework for other researchers to alter and improve. Moreover, there are scripts available at http://www.nitrc.org/plugins/mwiki/index.php/fcon_1000:ScriptUse to process this data and compute a variety of connectivity measures outside of the ICA-based measures presented here. These could be easily integrated with the methods outlined here to measure the connectivity properties once the connectivity matrices are established.

Although this analysis was created for analysis of fMRI data, more generally it applies to problems where the relationship among signal sources may determine the category to which an object belongs. The joint behavior of the signal sources (independent components) was observed as a graph object, where the distances between the sources represented the similarity of their behavior. Although second order measures were used to assess the functional connectivity (correlations), it is possible that as much discriminatory power exists using higher-order measurements that take into account the cohesiveness of triplets of components, or even more. Functional connectivity is one technique, of many, that should be assessed from multiple angles.

### Conflict of interest statement

The authors declare that the research was conducted in the absence of any commercial or financial relationships that could be construed as a potential conflict of interest.

## Appendix

### R functions and features tutorial

We begin by calling the library **AnalyzeFMRI** which must be pre-installed. When installing any package within the GUI, the option of “install dependencies” should be selected to install packages called by the main package. Once this package is installed, it is called, and the proper directory is navigated to. The current working directory can be obtained using getwd(). This directory needs to contain a list of scan names, filenames.txt, and the set of scans. We use here a scan produced by the FSL script supplied on the data available for download from NITRC.

> library(AnalyzeFMRI)
> rm(list = ls())
> setwd(“/path/to/my/directory”)

A scan can be read directly into *R*, and consequently held in memory, using

> myimage <- f.read.nifti.volume(“rest_mcf_brain.nii”)

We can get preliminary statistics using

> summary(myimage)
     Min.  1st Qu.  Median     Mean  3rd Qu.      Max.  
   0.0000   0.0000  0.0000 129.0000   0.0006 1639.0000

We can access the header information using

> f.nifti.file.summary(“rest_mcf_brain.nii”)

       File name: rest_mcf_brain.nii
  Data Dimension: 4-D
     X dimension: 64
     Y dimension: 64
     Z dimension: 33
  Time dimension: 150 time points
Voxel dimensions: 3.75 x 3.75 x 4.55000019073486
       Data type: (32 bits per voxel)

We can alternatively verify the dimensions of the scan using the command dim, since the image is held in memory.

> dim(myimage)
[1] 64 64 33 150

This image is stored as an array, which we can verify using

> is.array(myimage)
[1] TRUE

Functions such as f.read.nifti.volume, f.read.nifti.ts, and

f.read.nifti.slice.at.all.timepoints can allow direct access to specific pieces of data. For example, if we wanted to access a single timeseries from the 4D scan at voxel location (30,30,10) *without* holding the entire scan in memory, we can do so by:

> f.read.nifti.ts(“rest_mcf_brain.nii”,30,30,10)

where the input parameters are the filename and the (x,y,z) coordinates of the voxel desired. We can also access this from the scan held in memory using myimage[30,30,10,] which is noticeably faster.

We next wish to perform ICA within *R*, but this is available only for Analyze format scans. We write out our scan as Analyze format with:

> f.write.analyze(myimage,file=“rest_mcf_brain”)

If we wish to see all the parameter options available for ICA, we can access this using

> help(f.ica.fmri)}

We next run ICA on the example fMRI scan.

> g <- f.ica.fmri(“rest_mcf_brain.img”, n.comp=20, alg.type = “deflation”)

This performs ICA on the scan called “rest_mcf_brain.img”, extracting 20 components and storing the results in the object g. It is necessary to state the number of components to be extracted with the parameter ncomp. The number of components is somewhat arbitrary; the ICA extraction is performed here using the deflation approach where components are individually estimated and then subtracted out, so limiting the number of components theoretically should not change the structure of the components that are extracted. We set the default value, (num_components), to 20 according to (?). Use help(f.ica.fmri) to see the full list of options within the function

There is also the option of using a GUI to run this analysis, using the command f.ica.fmri.gui().

The object g contains several attributes:

> attributes(g)
$names
[1] “A“  “S”  “file” “mask”

The timeseries for the components are contained in g$A:

> dim(g$A)
[1] 150 20

The spatial maps are contained in the array g$S, the filename in g$file, and the mask (if used) in g$mask.

We can view a single component as shown in Figure 2, in this case the second of the set, by using

> f.plot.ica.fmri(g,2,cols=rainbow(100))

This image isn't thresholded, but can be roughly thresholded by adjusting the color options within rainbow(), by changing the number of colors to display to 3 and adjusting the saturation.

> f.plot.ica.fmri(g,2,cols=rainbow(3,alpha=.8))

We can also manually threshold using the spatial map g$S[, , ,2]. Here, we threshold the second component into the 10-th and 90-th percentile:

> g_thresholded <- g
> g_thresholded$S[,,,2][g$S[,,,2]>quantile(g$S[,,,2],probs=c(.1,.9))[2]] <- 3
> g_thresholded$S[,,,2][g$S[,,,2]<quantile(g$S[,,,2],probs=c(.1,.9))[2]] <- 2
> g_thresholded$S[,,,2][g$S[,,,2]<quantile(g$S[,,,2],probs=c(.1,.9))[1]] <- 1

The image can be written out using:

> f.write.analyze(g_thresholded$S[,,,2],file=“MyThresholdedImage”)

creating files called “MyThresholdedImage.img” and “MyThresholdedImage.hdr” in the working directory.

This concludes our example of the package **AnalyzefMRI**. This is not an exhaustive list of the functions available in this package, but a brief tutorial showing direct fMRI data access in R. For a full list of functions within this package, please see the manual at http://cran.r-project.org/web/packages/AnalyzeFMRI/AnalyzeFMRI.pdf.

### ICA for fMRI in R using preprocessed data

R can also perform ICA on Analyze format scans, so we demonstrate doing this in a loop. ICA within R uses the FAST-ICA algorithm, which is similar to FSL. This implementation will not work directly on the COBRE data since the function f.ica.fmri requires Analyze format data and the COBRE data is NIfTI, although the FSL command fslchfiletype will convert between the two formats. The filenames (“myfilename1.img”) are written on a text file called filenames.txt, where each line contains a separate filename. The lines that will need to be changed for the user are as follows:

> setwd(“/path/to/my/directory”)
> filenames <- read.table(“filenames.txt”)
> filenames <- filenames[i,1]
We can alternatively create the list of filenames using:
> filenames <- dir(pattern=“melodic_mix”)

We then create a loop to read in our files, perform ICA on each scan using the function f.ica.fmri() within the library **AnalyzeFMRI**, extract the timeseries associated with the components, and create a distance matrix for each component.

> library(AnalyzeFMRI)
> num_subjects <- dim(filenames)[1]
> num_components <- 30
> data_array_distance <- array(NA, c(num_subjects, num_components, num_components))
> my_distance <- function(x,y)
{  1-max(abs(ccf(x, y, plot = FALSE, lag.max = 3)$acf))  }

> for (i in 1:num_subjects)
{  #Perform ICA on each file
   temp <- f.ica.fmri(as.character(filenames[i,1]), n.comp = num_components,
                                                       alg.type=“deflation”)
   for(j in 1:num_components)
   {  j.vals <- rep(j,(num_components-j+1))
      k.vals <- (j:num_components)
      data_array_distance[i,j,j:num_components] <- c(mapply(x=j.vals,  
          y=k.vals, function(x,y) my_distance(temp$A[,x],temp$A[,y])))  
      data_array_distance[i,j:num_components,j]
                                <- data_array_distance[i,j,j:num_components] }
   diag(data_array_distance[i,,]) <- 0 }

This performs ICA on this *i*^th^ scan listed in filenames.txt, and stores the results into an object called temp. This object has several attributes, one of which is the timeseries associated with each component called temp$A.

### Parallel analysis in R

We now demonstrate parallel computing by using the base package **parallel** in R 2.1.4 as presented by http://www.bytemining.com/files/talks/larug/hpc2012/HPC_in_R_rev2012.pdf which provides an extensive explanation of parallel data analysis using *R*. The package **cudaBayesreg** also provides parallel analysis of fMRI data using multi-level Bayesian modeling.

The package **parallel** wraps together packages **multicore** and **snow**. We assume the computing is being done on the local machine here with multiple cores, but these methods can easily be extended to run on a cluster. The number of available cores can be detected using Windows will report the number of logical CPUs, which may exceed the number of physical cores.

> library(parallel)
> mc <- detectCores()
> mc
[1] 2

To perform the parallel analysis, we create a new function func_network_connectivity which takes as an argument the file number, and performs within each scan the FNC analysis.

> data_array_distance <- array(NA, c(num_subjects, num_components, num_components))
> num_components <- 20
> func_network_connectivity <- function(i){
    temp <- f.ica.fmri(as.character(filenames[i,1]), n.comp = num_components)
    for(j in 1:num_components)
    {   j.vals <- rep(j,(num_components-j+1))
        k.vals <- (j:num_components)
        data_array_distance[i,j,j:num_components] <-
             c(mapply(x=j.vals,y=k.vals, function(x,y)
             1-max(abs(ccf(x, y, plot = FALSE, lag.max = 3)$acf)))  
        data_array_distance[i,k.vals,j.vals] <- 1/data_array_distance[i,j,
             j:num_components]  }
    diag(data_array_distance[i,,]) <- 0
    d <- (data_array_distance[i,,])  
    my_iso <- isomap(d,axes=1, ndim = 10,epsilon=median(d),
           fragmentedOK=TRUE)
    my_net <- makegraph(my_iso) 
    d2 <- graph.adjacency(my_net, weighted = TRUE )  
    c(as.character(filenames[i,1]),average.path.length(d2),clique.number(d2),
         graph.density(d2),edge.connectivity(d2),median(closeness(d2)),
         median(graph.coreness(d2)),max(degree(d2)),median(degree(d2)),
         min(degree(d2)),vcount(d2),ecount(d2),transitivity(d2))    }

The new func_network_connectivity function is called by the parallel function parLapply, which is similar to mapply but operates in parallel. Using this, we perform ICA, establish functional connectivity and measure the graph structures in parallel. The function makeForkCluster is called to create multiple identical R processes on the same machine with a copy of the master workspace. This function will not work in Windows since Windows does not have a fork system call, so sockets must be used instead.

> cl <- makeForkCluster()
> clusterSetRNGStream (cl, 123)
> res <- parLapply(cl, seq_len(4), func_network_connectivity)
> stopCluster(cl)

## References

[B1] Achard S. Salvador R. Whitcher B. Suckling J. Bullmore E. (2006). A resilient, low-frequency, small-world human brain functional network with highly connected association cortical hubs. J. Neurosci. 26, 63–72 10.1523/JNEUROSCI.3874-05.2006 16399673 PMC6674299

[B2] Allen E. A. Erhardt E. B. Damaraju E. Gruner W. Segall J. M. Silva R. F. (2011). A baseline for the multivariate comparison of resting-state networks. Front. Syst. Neurosci. 5:2 10.3389/fnsys.2011.00002 21442040 PMC3051178

[B3] Anderson A. Bramen J. Douglas P. K. Lenartowicz A. Cho A. Culbertson C. (2011). Large sample group independent component analysis of functional magnetic resonance imaging using anatomical atlas-based reduction and bootstrapped clustering. Int. J. Imaging Syst. Technol. 21, 223–231 10.1002/ima.20286 22049263 PMC3204794

[B4] Anderson A. Dinov I. D. Sherin J. E. Quintana J. Yuille A. Cohen M. S. (2010). Classification of spatially unaligned fMRI scans. Neuroimage 49, 2501–2519 10.1016/j.neuroimage.2009.08.036 19712744 PMC2846648

[B5] Bassett D. S. Bullmore E. Verchinski B. A. Mattay V. S. Weinberger D. R. Meyer-Lindenberg A. (2008). Hierarchical organization of human cortical networks in health and schizophrenia. J. Neurosci. 28, 9239–9248 10.1523/JNEUROSCI.1929-08.2008 18784304 PMC2878961

[B6] Biswal B. Yetkin F. Z. Haughton V. M. Hyde J. S. (1995). Functional connectivity in the motor cortex of resting human brain using echo-planar MRI. Magn. Resona. Med. 34, 537–541 10.1002/mrm.1910340409 8524021

[B7] Bookheimer S. (2002). Functional MRI of language: new approaches to understanding the cortical organization of semantic processing. Annu. Rev. Neurosci. 25, 151–188 10.1146/annurev.neuro.25.112701.142946 12052907

[B8] Bordier C. Dojat M. de Micheaux P. L. (2011). Temporal and spatial independent component analysis for fMRI data sets embedded in the AnalyzeFMRI R package. J. Stat. Soft. 44, 1–24

[B9] Bordier C. Dojat M. de Micheaux P. L. (2009). **AnalyzeFMRI**: an *R* package to perform statistical analysis on fmri datasets. UseR 2009. Software: R Package, AnalyzeFMRI, version 1.1-12.

[B10] Calhoun V. D. Sui J. Kiehl K. Turner J. Allen E. Pearlson G. (2011). Exploring the psychosis functional connectome: aberrant intrinsic networks in schizophrenia and bipolar disorder. Front. Psychiatry 2:75 10.3389/fpsyt.2011.00075 22291663 PMC3254121

[B11] Chen G. Saad Z. S. Cox R. W. (2010). Statistical analysis programs in *R* for fMRI data, in UseR! 2010 Conference Proceedings. Available online at: http://www.r-project.org/conferences/useR-2010/abstracts/Chen+Saad+Cox.pdf

[B12] Chen G. Saad Z. S. Nath A. R. Beauchamp M. S. Cox R. W. (2012). FMRI group analysis combining effect estimates and their variances. Neuroimage 60, 747–765 10.1016/j.neuroimage.2011.12.060 22245637 PMC3404516

[B13] Chu C. Handwerker D. A. Bandettini P. A. Ashburner J. (2011). Measuring the consistency of global functional connectivity using kernel regression methods, in Proceedings of the 2011 IEEE International Workshop on Pattern Recognition in NeuroImaging (PRNI), 41–44

[B14] Cohen M. S. Bookheimer S. Y. (1994). Localization of brain function using magnetic resonance imaging. Trends Neurosci. 17, 268–277 10.1016/0166-2236(94)90055-8 7524210

[B15] Csardi G. Nepusz T. (2006). The **igraph** software package for complex network research. InterJournal Complex Systems, 1695

[B16] Dimitriadou E. Hornik K. Leisch F. Meyer D. Weingessel A. (2010). **e1071**: Misc Functions of the Department of Statistics (**e1071**), TU Wien. R package version 1.5-24.

[B17] Erdős P. Rényi A. (1961). On the strength of connectedness of a random graph. Acta Math. Hung. 12, 261–267 10.1007/BF02066689

[B18] Fan Y. Shen D. Davatzikos C. (2006). Detecting cognitive states from fmri images by machine learning and multivariate classification, in Proceedings of the Conference on Computer Vision and Pattern Recognition Workshop (CVPRW'06), 89–89

[B19] Ferreira da Silva A. R. (2011). CudaBayesreg: parallel implementation of a bayesian multilevel model for fMRI data analysis. J. Stat. Soft. 44, 1–24 10.1016/j.cmpb.2010.05.003 20580117

[B20] Ford J. Farid H. Makedon F. Flashman L. A. McAllister T. W. Megalooikonomou V. (2003). Patient classification of fMRI activation maps, in Medical Image Computing and Computer-Assisted Intervention-MICCAI (Berlin, Heidelberg: Springer), 58–65

[B21] Garrity A. Pearlson G. McKiernan K. Lloyd D. Kiehl K. Calhoun V. (2007). Aberrant default mode functional connectivity in schizophrenia. Am. J. Psychiatry 164, 450–457 10.1176/appi.ajp.164.3.450 17329470

[B22] Gauthier I. Tarr M. J. Anderson A. W. Skudlarski P. Gore J. C. (1999). Activation of the middle fusiform'face area'increases with expertise in recognizing novel objects. Nat. Neurosci. 2, 568–573 10.1038/9224 10448223

[B23] Georgiev P. G. Theis F. J. Cichocki A. (2005). Sparse component analysis and blind source separation of underdetermined mixtures. IEEE Trans. Neural Netw. 16, 992–996 10.1109/TNN.2005.849840 16121741

[B24] Hanlon F. M. Houck J. M. Pyeatt C. J. Lundy S. L. Euler M. J. Weisend M. P. (2011). Bilateral hippocampal dysfunction in schizophrenia. Neuroimage 58, 1158–1168 10.1016/j.neuroimage.2011.06.091 21763438 PMC4063352

[B25] Humphries M. D. Gurney K. (2008). Network small-world-ness: a quantitative method for determining canonical network equivalence. PLoS ONE 3:e0002051 10.1371/journal.pone.0002051 18446219 PMC2323569

[B26] Hyvärinen A. Oja E. (2000). Independent component analysis: algorithms and applications. Neural Netw. 13, 411–430 10.1016/S0893-6080(00)00026-5 10946390

[B27] Jafri M. J. Pearlson G. D. Stevens M. Calhoun V. D. (2008). A method for functional network connectivity among spatially independent resting-state components in schizophrenia. Neuroimage 39, 1666–1681 10.1016/j.neuroimage.2007.11.001 18082428 PMC3164840

[B28] Jenkinson M. Bannister P. Brady M. Smith S. (2002). Improved optimization for the robust and accurate linear registration and motion correction of brain images. Neuroimage 17, 825–841 10.1006/nimg.2002.1132 12377157

[B29] Kim S.-G. Rostrup E. Larsson H. B. Ogawa S. Paulson O. B. (1999). Determination of relative cmro 2 from cbf and bold changes: significant increase of oxygen consumption rate during visual stimulation. Magnet. Res. Med. 41, 1152–1161 10371447 10.1002/(sici)1522-2594(199906)41:6<1152::aid-mrm11>3.0.co;2-t

[B30] Koshino H. Carpenter P. A. Minshew N. J. Cherkassky V. L. Keller T. A. Just M. A. (2005). Functional connectivity in an fMRI working memory task in high-functioning autism. Neuroimage 24, 810–821 10.1016/j.neuroimage.2004.09.028 15652316

[B31] Lee T. M. Liu H.-L. Tan L.-H. Chan C. C. Mahankali S. Feng C.-M. (2002). Lie detection by functional magnetic resonance imaging. Hum. Brain Mapp. 15, 157–164 10.1002/hbm.10020 11835606 PMC6872015

[B32] Liang M. Zhou Y. Jiang T. Liu Z. Tian L. Liu H. (2006). Widespread functional disconnectivity in schizophrenia with resting-state functional magnetic resonance imaging. Neuroreport 17, 209–213 10.1097/01.wnr.0000198434.06518.b8 16407773

[B33] Liow J. S. Rehm K. Strother S. C. Anderson R. R. Mørch N. Hansen L. K. (2000). Comparison of voxel- and volume-of-interest-based analyses in FDG PET scans of HIV positive and healthy individuals. J. Nuclear Med. 41, 612–621 10.1093/brain/awn018 10768561

[B34] Liu Y. Liang M. Zhou Y. He Y. Hao Y. Song M. (2008). Disrupted small-world networks in schizophrenia. Brain 131, 945–961 18299296 10.1093/brain/awn018

[B35] Mayer A. R. Ruhl D. Merideth F. Ling J. Hanlon F. M. Bustillo J. (2012). Functional imaging of the hemodynamic sensory gating response in schizophrenia. Hum. Brain Mapp. 34, 2302–2312 10.1002/hbm.22065 22461278 PMC4020570

[B36] McKeown M. J. Hansen L. K. Sejnowsk T. J. (2003). Independent component analysis of functional mri: what is signal and what is noise? Curr. Opin. Neurobiol. 13, 620–629 10.1016/j.conb.2003.09.012 14630228 PMC2925426

[B37] Oksanen J. Blanchet F. G. Kindt R. Legendre P. O'Hara R. B. Simpson G. L. (2011). **vegan**: Community Ecology Package. R package version 1.17-6.

[B38] Polzehl J. Tabelow K. (2007). **fmri**: a package for analyzing fMRI data. RNews 7, 13–17

[B39] R Development Core Team. (2012). R: A Language and Environment for Statistical Computing. Vienna: R Foundation for Statistical Computing ISBN 3-900051-07-0.

[B40] Roebroeck A. Formisano E. Goebel R. (2005). Mapping directed influence over the brain using Granger causality and fMRI. Neuroimage 25, 230–242 10.1016/j.neuroimage.2004.11.017 15734358

[B41] Roelstraete B. Rosseel Y. (2011). FIAR: an R package for analyzing functional integration in the brain. J. Stat. Soft. 44, 1–32

[B42] Rubinov M. Sporns O. (2010). Complex network measures of brain connectivity: uses and interpretations. Neuroimage 52, 1059–1069 10.1016/j.neuroimage.2009.10.003 19819337

[B43] Sato J. R. Fujita A. Cardoso E. F. Thomaz C. E. Brammer M. J. Amaro E. (2010). Analyzing the connectivity between regions of interest: an approach based on cluster Granger causality for fMRI data analysis. Neuroimage 52, 1444–1455 10.1016/j.neuroimage.2010.05.022 20472076

[B44] Smith S. M. (2002). Fast robust automated brain extraction. Hum. Brain Mapp. 17, 143–155 10.1002/hbm.10062 12391568 PMC6871816

[B45] Smith S. M. Jenkinson M. Woolrich M. W. Beckmann C. F. Behrens T. E. J. Johansen-berg H (2004). Advances in functional and structural MR image analysis and implementation as *FSL*. Neuroimage 23, 208–219 10.1016/j.neuroimage.2004.07.051 15501092

[B46] Tabelow K. Clayden J. D. Lafaye de Micheaux P. Polzehl J. Schmid V. J. Whitcher B. (2011). Image analysis and statistical inference in neuroimaging with R. Neuroimage 55, 1686–1693 10.1016/j.neuroimage.2011.01.013 21238596

[B47] Tenenbaum J. B. de Silva V. Langford J. C. (2009). A global geometric framework for nonlinear dimensionality reduction. Science 290, 2319–2313 10.1126/science.290.5500.2319 11125149

[B48] Toppi J. De Vico Fallani F. Vecchiato G. Maglione A. Cincotti F. Mattia D. (2012). How the statistical validation of functional connectivity patterns can prevent erroneous definition of small-world properties of a brain connectivity network. Comput. Math. Methods Med. 2012 22919427 10.1155/2012/130985 PMC3420234

[B49] van de Ven V. G. (2004). Functional connectivity as revealed by spatial independent component analysis of fMRI measurements during rest. Hum. Brain Mapp. 22, 165–178 10.1002/hbm.20022 15195284 PMC6872001

[B50] Whitfield-Gabrieli S. Thermenos H. W. Milanovic S. Tsuang M. T. Faraone S. V. McCarley R. W. (2009). Hyperactivity and hyperconnectivity of the default network in schizophrenia and in first-degree relatives of persons with schizophrenia. Proc. Natl. Acad. Sci. U.S.A. 106, 1279–1284 10.1073/pnas.0809141106 19164577 PMC2633557

[B51] Yu Q. Sui J. Rachakonda S. He H. Gruner W. Pearlson G. (2011). Altered topological properties of functional network connectivity in schizophrenia during resting state: a small-world brain network study. PLoS ONE 6:e25423 10.1371/journal.pone.0025423 21980454 PMC3182226

[B52] Zalesky A. Fornito A. Bullmore E. (2012). On the use of correlation as a measure of network connectivity. Neuroimage 60, 2096–2106 10.1016/j.neuroimage.2012.02.001 22343126

[B53] Zhang L. Samaras D. (2005). Machine learning for clinical diagnosis from functional magnetic resonance imaging, in IEEE Conference on Computer Vision and Pattern Recognition (CVPR), (San Diego, CA), 1211–1217

